# Lysyl oxidase-like 4 involvement in retinoic acid epithelial wound healing

**DOI:** 10.1038/srep32688

**Published:** 2016-09-06

**Authors:** Aurélie Comptour, Marion Rouzaire, Corinne Belville, Nicolas Bonnin, Estelle Daniel, Frédéric Chiambaretta, Loïc Blanchon, Vincent Sapin

**Affiliations:** 1 Clermont Université, Université d’Auvergne, EA7281 – Retinoids, Reproduction Developmental Diseases, School of Medicine, F-63000 Clermont-Ferrand, France; 2 Clermont Université, Université d’Auvergne, GReD, F-63000 Clermont-Ferrand, France; 3 CHU Clermont-Ferrand, Ophthalmology Department, F-63000 Clermont-Ferrand, France

## Abstract

Vitamin A and its active forms (retinoic acids/RAs) are known to have pro-healing properties, but their mechanisms of action are still poorly understood. This work aimed to identify the cellular and molecular processes by which atRA (*all-trans* RA) improves wound healing, using an *in vivo* model of mouse corneal alkali burns and an *in vitro* cellular human corneal epithelial injury model. Regulation by atRA has been studied on most of the cellular events that occur in wound healing. We investigated the direct influence of atRA on a specific target gene known to be involved in the extracellular matrix (ECM) dynamics, one of the pathways contributing to epithelial repair. Our results demonstrate that atRA promotes corneal epithelial wound healing by acting preferentially on migration. The induction of lysyl oxidase-like 4 (LOXL4) expression by atRA in the corneal epithelium environment was established as essential in the mechanism of atRA-dependent wound healing. Our study describes for the first time a direct link between a retinoic-induced gene and protein, LOXL4, and its general clinical pro-healing properties in ECM dynamics.

Epithelial wound healing is a multistep combination of molecular and cellular events that have been extensively studied using epithelial models from skin, lung or cornea. Corneal epithelial wound repair is a complex dynamic process occurring after trauma, such as alkali burns, causing severe loss of visual acuity^1^. Comparable to the healing of skin epithelium, ocular wound repair involves important events such as migration, proliferation and differentiation of stem cells, as previously described^2^,^3^,^4^. Some studies have already been conducted to gain a fuller understanding of this process at the cornea level, using animal models and *in vitro* tests^5^. Corneal epithelial wound repair has been described as dependent on the dynamics of the extracellular matrix (ECM), which plays an important role in re-epithelialization by promoting cell adhesion and migration^6^,^7^. Of all the compounds already described as presenting pro-healing properties, vitamin A and its active derivatives (the retinoic acids or RAs) are among those most thoroughly studied. It is well established that these retinoids are essential in many physiological and developmental events from embryogenesis to adulthood^8^. In vision, they are required for eye morphogenesis and corneal integrity, by ensuring normal growth and differentiation of the epithelium layer^9^,^10^,^11^. Vitamin A deficiency (VAD) leads to ocular defects such as abnormal differentiation of ocular surface epithelium, resulting in keratinization and ulceration, superficial punctate keratitis and loss of conjunctival goblet cells^12^,^13^. Keratinization was reversed by an RA ointment treatment in a VAD xerophthalmic rabbit model^14^. Either as a nutrient intake or applied directly on the wound site surface, vitamin A and its active derivatives have already been shown to have a positive effect on wound healing^12^,^15^,^16^. Since the 1970s, its positive influence on corneal epithelium repair has been highlighted^17^,^18^,^19^,^20^,^21^,^22^,^23^. This signaling pathway has already been shown to be present and functional in the human corneal epithelium^24^.

The pleiotropic effects of RA are mediated by ligand-dependent nuclear receptors called respectively retinoic acid receptors (RARs) α, β and γ, and retinoid X receptors (RXRs) α, β and γ^25^,^26^. The transcriptional regulation of target genes was achieved by the binding of the heterodimer RAR/RXR to specific promoter sequences called retinoid acid response elements (RAREs) composed classically of two direct repeats of a hexameric motif (DR)^27^,^28^. The complete precise mechanism by which vitamin A and its principal active derivative (*all-trans* retinoic acid or atRA), promote wound healing remains poorly understood. Only a few genes have been characterized as both transcriptionally regulated by vitamin A and involved in various healing process, such as increase in hyaluronic acid or mucin (MUC16) production or modulation of pro-inflammatory cytokine production^17^,^29^. Moreover, recent studies describe one component of the plasminogen activator system, tissue type plasminogen activator (tPA) (retinoid-regulated gene involved in ECM dynamics)^30^, which must be precisely controlled for ideal wound healing of rat cornea^31^. Focusing on the ECM dynamics, the lysyl oxidase (LOX) family appears to be of particular interest for a full explanation of this process. The LOX family, a group of copper-dependent amine oxidase enzymes, is involved not only in ECM remodeling, but also in many biological functions in tumors or healthy tissues (migration, cell adhesion, metastasis^32^,^33^,^34^), where it initiates covalent crosslinking between component chains of collagen and elastin. The first identified LOX plays a critical role in ECM formation, stabilization and repair by contributing to the biogenesis of collagen and elastin^35^,^36^. Four other LOX-like proteins (LOXL^37^, LOXL2^38^, LOXL3^39^ and LOXL4^40^) have been described, sharing 95% homology between their C-terminal domains, containing the catalytic domain^41^. The members of the LOX family are essential, and must be precisely controlled: a deregulation of LOX expression has already been described in connective tissue diseases such as Menkes syndrome^42^ and in fibrotic diseases (arteriosclerosis)^43^. Concerning ocular disorders, the deregulation of LOX expression and activities is also the cause of keratoconus^44^, proliferative diabetic retinopathy and rhegmatogenous retinal detachment^45^. Among these LOX-linked disorders, previous results have also shown that in the case of keratoconus the healing process is remarkably inefficient^46^.

In this work, we characterize and demonstrate for the first time a cellular link between the pro-healing properties of vitamin A and a directly induced gene: LOXL4. These results, obtained for cornea wound healing treatment, open the way to a fully documented understanding of the positive effects of the vitamin A pathway, not only in the ocular sphere, but more generally in the clinical management of epithelial wound treatment.

## Results

### atRA is able to induce corneal wound healing on *in vivo* and *in vitro* models

**Figure 1:**
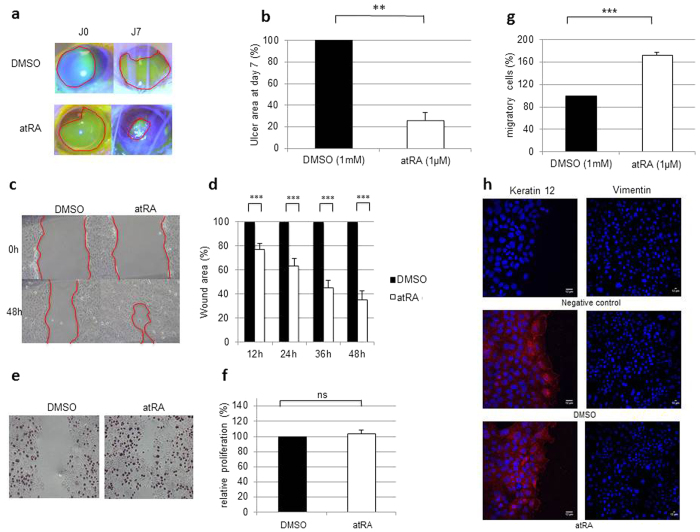
Induction by atRA of corneal wound healing on an *in vivo* and *in vitro* model. (**a**) Representative photographs of burned eyes stained with fluorescein. For each photograph, ulcer was delimitated by a red line. (**b**) Percentage of ulcer remaining after 7 days of treatment with atRA compared with DMSO (100%) on burned cornea (7 mice per group). The residual wound area obtained after DMSO treatment is fixed as 100%. (**c**) Representative images of scratch assay of HCE cells. For each image, wound area was delimitated by a red line. (**d**) Percentage of wound area of cells treated for 48 h with atRA compared with DMSO. The residual wound area obtained after DMSO treatment is fixed as 100% (*n* = 4 experiments each conducted in triplicate). (**e**) Representative images of brdU labeling on HCE cells. (**f**) Percentage of proliferating cells after 48 h of scratch assay on HCE cells treated with atRA compared with DMSO (100%), (*n* = 4 experiments each conducted in triplicate). (**g**) Percentage of migratory cells after 48 h of scratch assay on HCE cells treated with atRA compared with DMSO (100%) (*n* = 3 experiments each conducted in duplicate). (**h**) Keratin12 expression (cornea epithelial cell marker, in red) (left panel) and vimentin expression (right panel) in HCE cells scratch assay treated for 48 h by DMSO or atRA. Nuclei were counterstained with Hoechst (blue). For all graphs, each bar represents mean ± SD. Mann-Whitney *U*-test; **p < 0.01; ***p < 0.001; ns: not significant.

*In vivo* study models of wound healing showed that treatment of mouse corneal alkali burns with atRA (1 μM) for 7 days improved ulcer resorption, as seen by fluorescein staining (Fig. 1a). The percentage of remaining ulcer was only 26 ± 7% with atRA compared with the DMSO control group (100%) (Fig. 1b). *In vitro* scratch assay on HCE cells (Fig. 1c) confirmed that the wound area of cells treated with atRA shrank faster than that of DMSO-treated cells. From 12 h to 48 h (Fig. 1d), the percentage wound area between atRA- and DMSO-treated cells was significantly different at all four test points (12, 24, 36 and 48 h).

### atRA improves wound healing by acting principally on epithelial cell migration

By what cellular processes does atRA improve wound healing ? Migration and proliferation are the most classical events already described as important in this process, and experiments on HCE cells show that treatment with atRA increases the number of migrating cells by 72 ± 5.6% (Fig. 1g) compared with the DMSO condition. No significant difference was found for cell proliferation (Fig. 1e,f) after atRA treatment. To be sure that atRA did not affect the epithelial characteristics of HCE cells during the healing process, staining with keratin 12 (K12) (specific marker of the intermediate filaments in corneal epithelial cells^47^,^48^ was performed. Results (Fig. 1h, left panel) revealed no significant change in the distribution or intensity of the signal between the two treatment conditions (DMSO or atRA). No vimentin staining was detected after atRA treatment, ruling out epithelial-mesenchymal transition (Fig. 1h, right panel).

### LOXL4 is induced by atRA in HCE cells at transcript and protein levels

**Figure 2:**
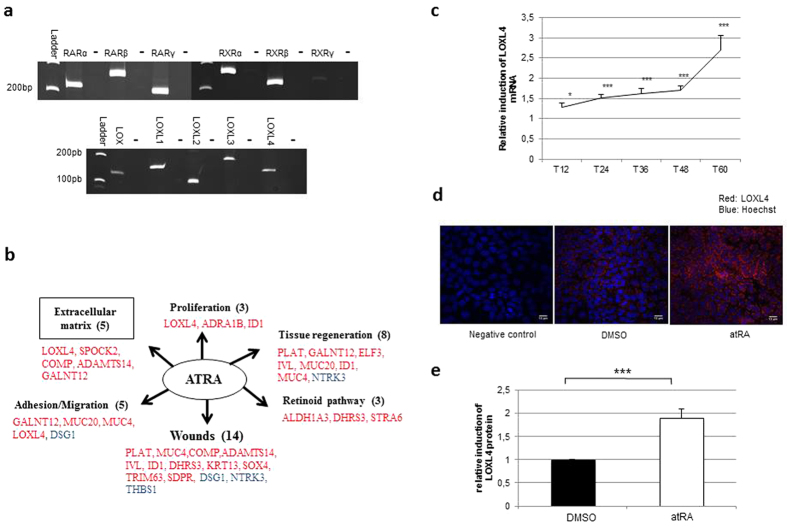
Retino-induction of LOXL4 gene expression and protein synthesis in HCE cells. (**a**) Characterization of the retinoic acid nuclear receptors RAR and RXR isoforms (α, β, γ) (top panel) and LOX family members (bottom panel) in HCE cells by PCR. Negative controls (−) were performed in the absence of cDNA. (**b**) The atRA-regulated genes identified by transcriptomic analyses on epithelial cells were classified according to their physiological pathways using Genomatix^®^. The most important of them are presented here with the number of genes belonging to each pathway indicated in brackets and listed below (atRA-induced genes are in red and atRA-repressed genes in blue). Abbreviations: ADAMTS14: ADAM metallopeptidase with thrombospondin type 1 motif, 14; ADRA1B: adrenergic, alpha-1B-, receptor; ALDH1A3: aldehyde dehydrogenase 1 family, member A3; COMP: cartilage oligomeric matrix protein; DHRS: dehydrogenase/reductase (SDR family) member 3; DSG1: desmoglein 1; ELF3: E74-like factor 3; IVL: involucrin; GALNT12:UDP-*N*-acetyl-alpha-D-galactosamine: polypeptide *N*-acetylgalactosaminyl-transferase 12; ID1: inhibitor of DNA binding 1, dominant negative helix-loop-helix protein; KRT13: keratin 13; LOXL4: lysyl oxidase-like 4; MUC4: mucin 4; MUC20: mucin 20; NTRK3: neurotrophic tyrosine kinase, receptor, type 3; PLAT: plasminogen activator, tissue; SDPR: serum deprivation response; SOX4: SRY (sex determining region Y)-box 4; SPOCK2: testican 2; STRA6: stimulated by retinoic acid gene 6; THBS1: thrombospondin 1; TRIM63: tripartite motif-containing 63. (**c**) LOXL4 RNA induction in HCE cells treated for 12, 24, 36, 48 and 60 h with atRA normalized by the geometric mean of three housekeeping genes (*n* = 5 experiments each conducted in duplicate). (**d**) Representative pictures of LOXL4 expression (red) in HCE cells treated for 48 h with atRA. Nuclei were counterstained with Hoechst (blue). (**e**) LOXL4 protein synthesis quantified by immunofluorescence on HCE cells treated for 48 h with atRA and expressed as a ratio to DMSO (*n* = 3 experiments each conducted in triplicate, 3 quantifications per slide). Each bar shows mean ± SD. Mann-Whitney *U*-test; *p < 0.05; ***p < 0.001.

To clarify the atRA wound healing action, we first identified the presence of all the isoforms (α, β, γ) of the retinoic nuclear receptors RAR and RXR in the cellular model of HCE (Fig. 2a, upper panel). Based on targeted transcriptomic results obtained to design retinoic acid target genes potentially involved in wound healing, we classified them according to their physiological pathways using the genomatix program. We identified the LOX family as strongly involved with its presence for 3 out of 6 identified pathways (Fig. 2b). PCR analysis demonstrated that all LOX-like genes were expressed in HCE cells (Fig. 2a, lower panel). Nevertheless, RT q-PCR experiments on HCE cells treated with atRA showed that only LOXL4 was induced from 12 h (1.28 ± 0.1) to 60 h (2.71 ± 0.35) by atRA treatment (Fig. 2c and Supplementary Data 1 for LOX, LOXL1, LOXL2 and LOXL3). Transcript results were confirmed at the protein level by immunocytochemistry experiments (confirmed by ELISA assays, Supplementary Data 2), where protein accumulation was increased as illustrated by a factor of 1.9 ± 0.2 after 48 h of treatment (Fig. 2d,e).

### atRA induction of LOXL4 gene transcription involves RARα fixation on one DR5 site

**Figure 3:**
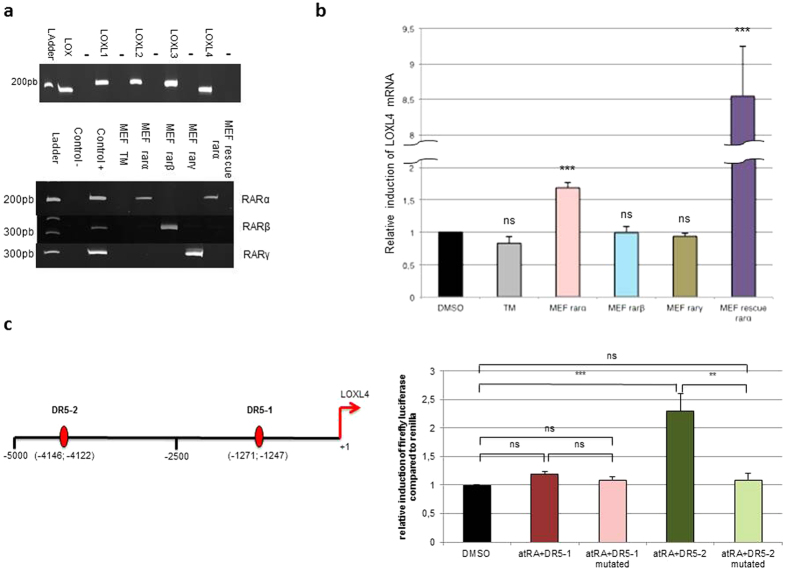
Involvement of RARα in the induction of LOXL4 transcription by its fixation on a single DR5-RARE binding site. (**a**) Characterization of the LOX family member’s expression in TM (triple mutants) MEF cells (top panel). Characterization of the retinoic acid nuclear receptor RAR isoform (α, β, γ) expression (bottom panel, left side) in native and transiently transfected (by RARα, by RAR β or by RAR γ expression plasmid). Confirmation of RARα expression in TM rescue RARα MEF cells (bottom panel, right). For each experiment, negative controls (control −) was performed in the absence of cDNA, positive controls represent cDNA from mouse cornea. (**b**) LOXL4 RNA expression is induced in TM MEF cells transiently transfected with RARα and in TM rescue RARα MEF cells treated for 12 h with atRA normalized by the geometric mean of three housekeeping genes (*n* = 3 experiments each conducted in duplicate) (Mann-Whitney *U*-test; ***p < 0.001; ns: not significant). (**c**) HCE cells transiently transfected with constructs of LOXL4 gene promoter (DR5-1, mutated DR5-1, DR5-2, mutated DR5-2/see schematic representation of the LOXL4 gene promoter) and RARα/RXRα expression vectors, and treated with atRA for 48 h. Luciferase quantification is normalized using dual luciferase system, and expressed as fold induction relative to DMSO treatment (*n* = 3 experiments each performed in duplicate) (Kruskal-Wallis test; **p < 0.01; ***p < 0.001; ns: not significant). Each bar shows mean ± SD.

To identify the nuclear retinoid actors involved in this LOXL4 regulation, triple mutant (RARα, RARβ, and RARγ) and RARα rescue MEF cells were used. The expression of the different LOX family members was first demonstrated (Fig. 3a, top panel) in this cellular model. To determine which isoforms of RAR were involved in the activation of LOXL4, transient transfections of RARα, β or γ triple mutant cells were carried out (Fig. 3a, bottom panel), which showed that only the RARα transfection could lead to an atRA induction of LOXL4 expression (1.7 ± 0.1). This result was confirmed by the use of TM rescue RARα MEF cells, where the induction of LOXL4 by atRA was present and stronger (8.5 ± 0.7) (Fig. 3b). Bio-informatics studies using Genomatix^®^ software highlighted the presence of two DR5^49^ in the promoter of LOXL4: DR5-1 (−1247 to −1271 pb) and DR5-2 (−4122 to −4146 pb) (Fig. 3c, left panel). Transient transfection in HCE cells of both constructs (DR5-1 and DR5-2) demonstrated after atRA treatment that DR5-2 (induction factor of 2.3 ± 0.3) seemed to be involved in the atRA-dependent regulation of LOXL4 transcription. This result was confirmed by mutagenesis studies, showing a total loss of LOXL4 induction when the DR5-2 was mutated, whereas no change was detected for DR5-1 (Fig. 3c, right panel).

### LOXL4, induced by atRA is essential for *in vitro* wound healing of HCE cells

**Figure 4:**
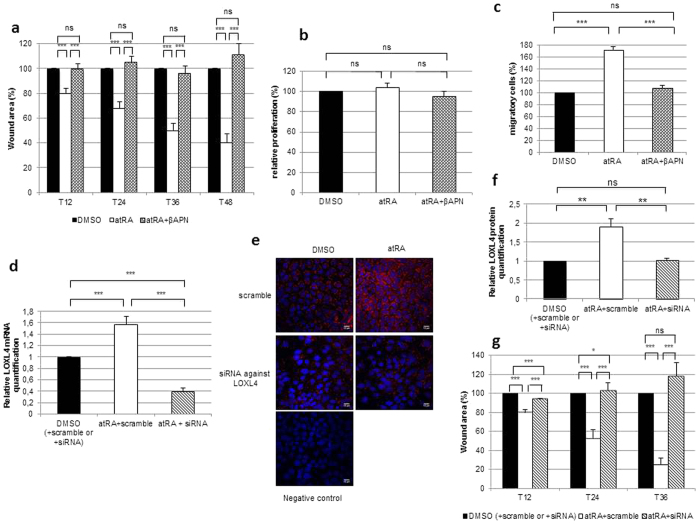
Decrease in the wound healing process promoted by atRA by enzymatic or post-transcriptional inhibition of LOXL4. (**a**) Percentage of wound area of cells treated for 48 h with atRA or atRA + βAPN for 48 h compared respectively with DMSO or DMSO + βAPN. The residual wound area obtained after DMSO treatment is fixed as 100% (*n* = 4 experiments each conducted in triplicate) (***p < 0.0001). (**b**) Percentage of proliferating cells after 48 h of scratch assay on HCE cells treated with atRA or atRA + βAPN compared respectively with DMSO or DMSO + βAPN (100%) (*n* = 4 experiments each conducted in triplicate). ns: not significant. (**c**) Percentage of migratory cells after 48 h of scratch assay on HCE cells treated with atRA or atRA + βAPN compared respectively with DMSO or DMSO + βAPN (100%) (*n* = 3 experiments each conducted in duplicate) (***p < 0.001). (**d**) LOXL4 mRNA extinction in HCE cells transiently transfected by a siRNA control (scramble) or siRNA against LOXL4 and treated for 48 h with atRA. Results were normalized by the geometric mean of three housekeeping genes, and expressed as a ratio to DMSO (*n* = 3 experiments each conducted in duplicate) (***p < 0.001). (**e**) Representative pictures of LOXL4 expression (red) in HCE cells transiently transfected by a siRNA scramble or siRNA against LOXL4 and treated for 48 h with atRA. Nuclei were counterstained with Hoechst (blue). (**f**) LOXL4 protein extinction quantified by immunofluorescence on HCE cells transiently transfected by a siRNA scramble or siRNA against LOXL4 and treated for 48 h with atRA and expressed as a ratio with DMSO (*n* = 3 experiments each conducted in triplicate, 3 quantifications per slide) (**p < 0.01). (**g**) Percentage of wound area of cells transiently transfected by a siRNA control or a siRNA against LOXL4 and treated for 48 h with atRA compared respectively with DMSO condition (*n* = 3 each experiment with triplicate samples). The residual wound area obtained after DMSO treatment is fixed as 100% (***p < 0.001). Each bar shows mean ± SD. ns: not significant.

Involvement of LOXL4 in the wound-healing process promoted by atRA was investigated using the scratch assay technique and a well-known enzymatic inhibitor of the LOX family, βAPN (*β*-aminopropionitrile). In agreement with the literature^50^,^51^, we first found that a concentration of 500 μM was not toxic for the corneal cell model by obtaining stability of LDH (lactate dehydrogenase) concentrations in culture media (Supplementary Data 3). Treatment of HCE cells by atRA or a combination of atRA + βAPN clearly showed that the significant difference in the percentage of wound healing between DMSO- and atRA-treated cells disappeared when βAPN was used in combination with atRA. This was illustrated at 48 h of scratch assay, with the percentage of wound area equal to 100% for vehicle, 40 ± 7% for atRA treatment and 111 ± 9% for cells treated with atRA + βAPN (Fig. 4a). The influence of LOX enzymatic inhibition was then tested on cell proliferation and migration as depicted in Fig. 1f,g. No difference in the percentage of proliferating cells could be observed between cells treated with vehicle and cells treated with atRA, or with cells treated with βAPN (Fig. 4b). On the other hand, βAPN treatment clearly prevented cell migration, as shown in Fig. 4c (108 ± 5% against 172 ± 6% for cells treated with atRA + βAPN and atRA respectively).

Because βAPN is not specific to any one member of the LOX family, the specific involvement of LOXL4 in wound healing was further confirmed by a siRNA technique. First, the efficiency of siRNA transfection was tested on LOXL4 expression at transcript (affecting induced and endogenous) and protein levels. In detail, RT-qPCR experiments showed that in cells transfected by siRNA scramble, LOXL4 was induced by atRA by a factor of 1.6 ± 0.1 at 48 h of scratch assay, but in cells transfected by siRNA against LOXL4, the expression of LOXL4 was strongly reduced (Fig. 4d). At protein level, immunochemistry experiments revealed that in cells transfected by siRNA scramble, LOXL4 staining was stronger for cells treated with atRA and strongly reduced for cells transfected with siRNA against LOXL4 (Fig. 4e). Negative control of gene extinction by siRNA against LOXL4 was also done at the mRNA levels for atRA induced genes (RARβ and STRA6) and for a non-aTRA induced gene member of the lysyl oxidase family (Supplementary Data 4). Quantification of LOXL4 staining (Fig. 4f) confirmed an induction of LOXL4 (1.9 ± 0.2) by atRA in cells transfected by siRNA scramble, and a reduction of LOXL4 accumulation protein (1.0 ± 0.1) when cells were transfected by a siRNA against LOXL4. A scratch assay was carried out in the presence of this siRNA (Fig. 4g), which showed that wound closure for cells transfected with siRNA against LOXL4 reverted to the level found for DMSO treatment (between 94 ± 1% for 12 h and 118 ± 14% for 36 h).

### LOXL4, induced by atRA, is essential for *in vivo* wound healing of mouse cornea

**Figure 5:**
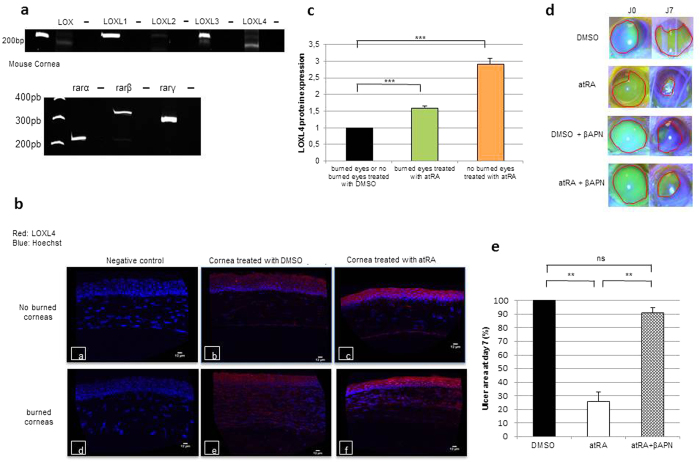
LOXL4 protein induction by atRA to promote corneal wound healing on an *in vivo* mouse model. (**a**) Characterization of the LOX family member’s expression (top panel) and retinoic acid nuclear receptors RAR isoforms (α, β, γ) (bottom panel) in mouse cornea by PCR. Negative controls (−) were performed in the absence of cDNA. (**b**) Representative pictures of LOXL4 expression (red) in unburned corneas (**a**,**b**,**c**) and burned corneas (**d**,**e**,**f**) treated with DMSO (**b**,**e**) or atRA (**c**,**f**). The nuclei were counterstained with Hoechst (blue); negative controls (**a**,**d**) were obtained without primary antibody incubation. (**c**) Quantification of LOXL4 epithelium staining on unburned and burned corneas treated with atRA. All results are expressed as a ratio with DMSO (*n* = 3 quantifications per slide, 4 mice per group). (**d**) Representative pictures of burned eyes stained with fluorescein. For each photograph, ulcer was delimitated by a red line. (**e**) Percentage of ulcer remaining after 7 days of treatment with atRA or atRA + βAPN (500 μM) compared with DMSO (100%) on burned corneas (7 mice per group). The residual wound area obtained after DMSO treatment is fixed as 100%. Bars show mean ± SD. Mann-Whitney *U*-test; **p < 0.01; ***p < 0.001, ns: not significant.

Going back to our *in vivo* mouse model of wound healing, the major role that LOXL4 plays in this process was investigated. Using PCR, we demonstrated the expression of all LOX-like genes (Fig. 5a, top panel), and all the RAR isoforms (α, β, γ) (Fig. 5a, bottom panel) in mouse cornea. Thus to find out whether LOXL4 was induced by atRA at the protein level in mouse cornea, unburned or burned corneas treated with atRA (or vehicle DMSO) were analyzed by immunohistochemistry. When corneas, burned or unburned, were treated with atRA, a strong accumulation of LOXL4 protein appeared specifically in the epithelium zone (Fig. 5b,c). Lastly, the same experiments on mice were conducted as previously described, but in the presence or absence of βAPN. They confirmed that LOXL4 was essential for the wound healing process promoted by atRA. As illustrated in Fig. 5d, fluorescein staining of the ulcer surface demonstrated that the percentage of ulcer remaining after 7 days of treatment was still near 90% for eyes treated with atRA + βAPN, against around 26% for eyes treated with atRA (Fig. 5e).

## Discussion

Corneal wound healing is an essential clinical step in obtaining the best possible recovery of visual acuity after, for example, eye trauma caused by ocular chemical burns. Our study focused on the effect of atRA on this mechanism using *in vivo* and *in vitro* approaches. First, we showed on a mouse model of alkali burns that atRA was able to promote wound healing as previously demonstrated on other animal models and by using vitamin A and/or its active derivatives^17^,^18^,^19^,^20^. Switching to an *in vitro* model of wounded HCE cells enabled us to investigate the still-unknown cellular and molecular processes by which atRA acts. Migration, proliferation and differentiation are the most important and best-described events in epithelial wound repair. For the first time, atRA was shown to act preferentially on migration rather than epithelial proliferation and differentiation to stimulate wound healing. These results agree with those obtained by Hattori *et al.*, but extend our knowledge of atRA influence by underlining the importance of the migration process. These authors have already demonstrated in a model of HCE-T cells that atRA combined with nanoparticle vehicles, used at high concentration, has a positive effect on wound healing, with no effect on cell proliferation or differentiation^18^.

Tissue remodeling and especially cell migration are dependent on the balance between the destruction and formation of ECM and its ability to mediate cell adhesion^52^,^53^. The link between atRA regulated genes and ECM dynamics and remodeling has already been described in several tissues, *e.g.* the skin^54^,^55^. For the eye, it has been shown that a treatment with atRA on corneal stromal keratinocytes cultured *in vitro* increases the production of ECM components such as collagen type 1, and reduces the expression of matrix metalloproteinase (MMP)-1, MMP-3 and MMP-9 (responsible for the disintegration of the ECM)^56^. Interestingly, it has been demonstrated that MMP9 is essential in the wound healing process for regulating re-epithelialization and migration of cells^17^,^57^, and that an abnormal elevation of its amount is correlated with abnormal re-epithelialization in other tissues^58^. In addition, and again in tissues other than ocular, it has been demonstrated that atRA influences overall balance rather than only ECM degradation, as seen for example in the MMP-2/TIMP-2 ratio in a model of cultured human fetal palate mesenchymal cells^59^. In our study, to describe the direct relation between a vitamin A regulated gene and wound healing, we opted to focus on another component of ECM remodeling, the lysyl oxidase family, which plays a crucial role by acting on collagen biosynthesis^60^. This choice was made because the deregulation of these oxidases leads to severe connective tissue disorders and ocular diseases such as keratoconus^42^,^43^,^44^. We demonstrate that one of the LOX-like proteins, LOXL4, is directly induced by atRA at transcript and protein levels in HCE cells and mouse corneas. In this paper, this positive transcription of LOXL4 by retinoic acid is described for the first time. In the LOX family, only LOX and LOXL2 were already established as retinoic acid target genes, but in tissue environments other than corneal^38^, demonstrating the great plasticity of retinoic acid as a gene regulator.

The LOXL4 regulation by atRA involved nuclear retinoic acid receptor RARα fixation on the second DR5 in LOXL4 promoter (−4122, −4146 pb). Furthermore, we found that this gene was essential for corneal wound healing promoted by atRA on HCE cells and on a mouse model of corneal injury by promoting cell migration. LOXL4 regulation by atRA presents the twofold advantage of positively regulating one molecular step (ECM reinforcement with its action on collagen) and one cellular step (epithelial migration). This combined effect of atRA on the corneal environment, added to others previously reported, could synergize in wound healing, reversing corneal keratinization, preserving epithelial barrier function or increasing stromal keratocyte number *in vitro*^9^.

Ocular injury is found in approximately 15–20% of patients with facial burns^61^. Among the ocular injuries, ocular chemical burns represent some 10% of eye trauma^62^,^63^. Understanding the pathophysiology of these injuries is essential for improving the management of corneal burns. Several studies on animal models have already demonstrated the positive effect of retinoic acid on corneal wound healing by preventing harmful events, such as neovascularization and opacity of the cornea^64^,^65^. By describing a gene by which atRA and more generally vitamin A permit wound healing, this work opens the way to the study and direct targeting of other genes involved in ECM formation and remodeling. Our findings could lead on to future clinical therapies using vitamin A alone or in combination with other medications and already long-used surgical treatment, such as amniotic membrane grafts^66^,^67^.

## Methods

### Mice and *in vivo* model of corneal epithelial wound healing

Mice were maintained and procedures were performed with the approval of the Auvergne Regional Ethics Committee (CEMEA Auvergne) in the animal facility of the School of Medicine – University of Clermont-Ferrand (approval No. 63.113.15). All the experiments were conducted in accordance with the ARVO Resolution for the Use of Animals in Ophthalmic and Vision Research. Twenty-eight white male CD1 mice aged 4–6 weeks were used. At Day 0, under general anesthesia with intraperitoneal injection of pentobarbital (0.82 mg), a standardized chemical corneal burn was performed by placing a filter paper (circular, diameter 3.0 mm) saturated with NaOH (1 N) for 15 s on the sclero-corneal limbus. The wound surface was then washed with Balance Salt Solution^®^, and antibiotic (norfloxacin: Chibroxine^®^, 0.3% eye drops) was applied on it three times per day. Finally, each injured cornea was treated with eye drops six times daily for 7 days. Mice were divided into four groups of seven mice receiving a treatment with atRA (1 μM) (Sigma) dissolved in dimethyl sulfoxide (DMSO/atRA vehicle) (Sigma) or with DMSO (0.1%) (control group) or with DMSO and βAPN (ß-aminopropionitrile) (500 μM) (Sigma) or with atRA (1 μM) and βAPN (500 μM). Same dilution ratio (0.1%) for DMSO was always realized in physiological serum. Wound size was determined by staining with fluorescein (0.5%) and photographing at Days 0 and 7. Wound area was quantified from photographs using imageJ V.1.31. Unburned or burned corneas treated with DMSO or atRA were collected and stored frozen at −80 °C for PCR experiments, or cryopreserved in OCT for immunohistochemistry.

### Cell cultures

Human corneal epithelial (HCE) cells transformed with SV40 (ATCC/CRL11135) were cultured under standard conditions (5% CO_2_, 95% humidified air, 37 °C) as previously described^24^. Triple mutant (RARα, RARβ, RARγ) MEF (mouse embryonic fibroblast) cells and (constitutively) rescue RARα MEF cells (obtained from Prof Hugues de Thé (Université Paris Diderot, Paris, France)) were cultured in Dulbecco’s Modified Eagle Medium (DMEM) (Gibco) supplemented with 10% fetal bovine serum l-glutamine solution (2 mM) (Gibco) and 50 mg/mL of steptomycin, 50 IU/mL of penicillin.

### Transcriptomics study

RNA was extracted and quality-controlled using the Agilent RNA 6000 Nano Kit. cDNA was then obtained using the Superscript III First-Strand-Synthesis System for RT-PCR. The cDNA was hybridized on a Human genome 8 × 60 K transcriptomics chip (Agilent, Santa Clara, CA) by a local lab (Hybrigenics, Saint-Beauzire, France). Genes were classified according to fold change and biological pathway using the Genomatix^®^ pathway system (GePS). The results were furnished by the company after this bioinformatics analysis as a genes list containing gene name, gene abbreviation, log fold change and accession number.

### *In vitro* model of corneal epithelial wound healing (scratch assay)

Confluent HCE cells placed in four-well plates were manually scraped with a pipette tip. After several washings with PBS (1X) (Gibco), wounded cells were treated with DMSO (0.1%) (control group) or with atRA (1 μM) or with DMSO (0.1%) and βAPN (500 μM) or with atRA (1 μM) and βAPN (500 μM) every 24 h for 48 h. Same dilution ratio (0.1%) for DMSO was always used in culture media. Pictures of the wound were taken every 12 h under a microscope, and wound areas were measured using imageJ software every 12 h for 48 h.

### Cell proliferation and migration assay

BrdU staining was conducted using a 5-bromo-2′-deoxy-uridine labeling and detection Kit II (Roche) according to the manufacturer’s instructions. The percentage of proliferating cells for each condition was calculated by the ratio of BrdU positive cells to the total number of cells. Cell migration was assessed with a CytoSelect™ 24-well cell migration assay (8 μm, fluorometric format) (Biolabs) according to the manufacturer’s instructions. Migratory cells were stained with CyQuant^®^ GR Dye (Invitrogen) and quantified by a fluorescence measurement at 480 nm/520 nm. This experiment was repeated three times (each condition in duplicate).

### Enzyme-linked immunosorbent assay

ELISA assay was assessed using a commercial LOXL4 ELISA kit (DL-LOXL4-Hu; Wuxi Donglin Sci and Tech Development Co. Ltd) on HCE cell lysates according to the manufacturer’s instructions. Absorbance of each sample was measured at 450 nm. The concentration of LOXL4 in the samples was then determined by comparing the absorbance of the samples with the standard curve. Results were normalized by measuring the total protein concentration in each sample using a BSA protein assay kit (Pierce). Each sample was loaded on the microplate in duplicate.

### Immunohistochemistry and immunocytochemistry experiments

Immunohistochemistry and immunocytochemistry experiments were performed on cryosections (10 μm) of mouse corneas (four mice per group) and on HCE cells after 48 h of treatment with atRA or DMSO respectively. Slides were fixed with paraformaldehyde (4% in PBS) and incubated overnight with a rabbit polyclonal anti-LOXL4 antibody (1/200) (ab88186; Abcam) (slides of tissues and cells) or with a mouse monoclonal anti-vimentin antibody (1/40) (090M4817; Sigma-Aldrich) or a goat polyclonal anti-keratin 12 (1/50) (L-20 sc-17099) for slides of cells. Slides of tissues and cells were then respectively incubated with a goat anti-rabbit antibody (Jackson) or anti-mouse (Life Technology) antibodies conjugated with Alexa488 or donkey anti-goat conjugated with cyanine 3 (Jackson) for 2 h. Nuclei were counterstained with Hoechst (bisBenzimide H 33258) (Sigma) (1/10000) for 10 min at RT. Finally, slides were examined under fluorescent microscopes (Zeiss Axiophot or Leica SPE5). Control samples were obtained without the primary antibody, and after incubation of primary antibody with blocking peptide. For induction of LOXL4 protein, quantification of LOXL4 staining was performed using imageJ and, in cells, was confirmed by ELISA test (Supplementary Data 2).

### Plasmid constructions

For LOXL4 promoter analysis, the different constructs (DR5-1; DR5-2) were obtained using PCR amplifications from BAC (RPCI-11 HS) (Fisher) (see Table 1), and cloned into the pGL3 basic vector containing *firefly* luciferase (pGL3b) (Promega). The mutagenesis of DR5 sites (four mutated bases in the core sequence) into DR5-1-pGL3b or DR5-2-pGL3b constructs was carried out by PCR (see Table 1). All the constructions were verified by DNA sequencing (GATC). Human RAR (α, β, γ) and RXRα expression plasmids were obtained from Pierre Chambon (IGBMC, Strasbourg, France). Co-transfection of pRL-tk vector (1/20) (Promega) containing *Renilla* luciferase allowed the normalization of the transfection efficiency.

### Cell transfections

LOXL4 expression was depleted in HCE cells using a small interfering RNA (siRNA) technique. First, HCE cells placed in six-well plates were treated with atRA or DMSO for 24 h. For transfection, 1 μL of a commercial siRNA (10 μM) (SI00142450; QIAGEN) against LOXL4, or 1 μL of a nonspecific siRNA (10 μM) (siRNA scramble; SI03650318; QIAGEN) in 125 μL of optiMEM^®^I (1X) medium (Gibco), was mixed with 2.5 μL of lipofectamine^®^ RNAiMAX reagent (Invitrogen) in 125 μL of optiMEM^®^I (1X) and incubated for 20 min before transfection in cells. Cells were treated with atRA or vehicle DMSO during the transfection, and for 48 h afterwards. For LOXL4 promoter analysis, HCE cells placed in six-well plates were transiently transfected with 0.5 μg of each human RARα and RXRα expression plasmids, 1 μg of different LOXL4 promoter constructs, and 0.05 μg of pRL-tk vector with 6.4 μL of nanofectin reagent (PAA). After incubation overnight, the cells were treated for 48 h with atRA or DMSO. Finally, luciferase activity was measured using the Dual-Luciferase^®^ reporter assay system (Promega) according to the manufacturer’s instructions. Triple mutant (RARα, RARβ, and RARγ) and rescue RARα MEF cells were transiently transfected in six-well plates with 0.5 μg of each human RARα, β or γ and RXRα expression plasmids in the presence of 3.2 μL of nanofectin reagent, and simultaneously treated with atRA or DMSO for 48 h. These transfections were repeated three times in duplicate.

### Quantitative RT-PCR and PCR experiments

Total RNA was extracted from mouse corneas and cell cultures using RNeasyMini Kit (Qiagen). For cDNA synthesis, 1 μg of RNA was reverse-transcribed using a Superscript III First-Strand Synthesis System for RT-PCR (Invitrogen). Quantitative RT-PCR reactions were performed using SensiFAST**™**SYBR (Bioline) and Light Cycler^®^480 SYBR Green I Master (Roche). Transcripts were quantified independently three times in three independent experiments, and normalized by the geometric mean of three housekeeping genes (RPLP0, 18S rRNA and *β*-actin). All the steps followed the MIQE guidelines. Primers used for PCR and qPCR are detailed in Table 1.

### Statistics

The data expressed as means ± SDs are the average of duplicates or triplicates of three or four independent experiments. Comparison of means was performed by a nonparametric *U*-test (Mann-Whitney) or Kruskal-Wallis test using GraphPad PRISM software (GraphPad Software Inc.). Throughout, values were considered significantly different at *p* < 0.05.

## Additional Information

**How to cite this article**: Comptour, A. *et al.* Lysyl oxidase-like 4 involvement in retinoic acid epithelial wound healing. *Sci. Rep.* **6**, 32688; doi: 10.1038/srep32688 (2016).

## Supplementary Material

Supplementary Information

## Figures and Tables

**Table 1:** Sequences of oligonucleotides used for RT-PCR, RT-qPCR, cloning and mutagenesis.

genes	sequence (5′-3′)		Product size (bp)	Accession n°
	Forward	reverse		
human LOX	GATACGGCACTGGCTACTTCC	CTGGCCAGACAGTTTTCCTCC	136	NM_002317.5
human LOXL1	AGCATCCACTTATGTGCAGAGA	GAGGAAGTCTGCTGTGCCCT	166	NM_005576.2
human LOXL2	CACTGCGGATCCCTGAAACC	CTGTCTTCGGGCTGATGATCC	103	NM_002318.2
human LOXL3	CAGGCTGCCCACATCCTCTG	ATCCTCATCGTGCGTACAGTC	189	NM_032603.3
human LOXL4	Commercial (QIAGEN)		146	NM_032211.6
human RARα	AGTCCTCAGGCTACCACTAT	CCTCCTTCTTCTTCTTGTTT	225	NM_000964.3
human RARβ	ATGGATGTTCTGTCAGTGAG	CATAGTGGTACCCTGATGAT	268	NM_000965.4
human RARγ	ACCAATAAGGAGCGACTCT	ATCTCCTCTGAGCTGGTG	212	NM_000966.5
human RXRα	GGATCCCACACTTCTCAG	GAGTCAGGGTTAAAGAGGAC	286	NM_002957.5
human RXRβ	AGTACTGCCGCTATCAGAA	GTTAGTCACAGGGTCATTTG	242	NM_001270401.1
human RXRγ	CTACACAGATACCCCAGTGA	GGGTAGTTCATGTTTCCAAT	249	NM_006917.4
human RPLP0	AGGCTTTAGGTATCACCACT	GCAGAGTTTCCTCTGTGATA	219	NM_001002.3
mouse LOX	CATAGATCGCATGGTGGGCG	ACTACATCCAGGCTTCCACGTA	195	NM_010728.3
mouse LOXL1	CGTGCTGGAGCCACCTTACT	TCCTTGCGATGCGCAGCAGA	219	NM_010729.3
mouse LOXL2	GCACACTGAAGACGTTGGAGT	AAGGCTTGGAAGCAGATCTGC	208	NM_033325.2
mouse LOXL3	GATGATGACTTCACGCTGCAG	GTGACTGTACCCATGATGAGG	205	NM_013586.4
mouse LOXL4	CCAAGTATGGTCAAGGAGAGG	AAGGTCTCCAATGCCCTCGG	193	NM_001164311.1
mouse RARα	CACCTCAATGGGTACCCAGTA	AAGCAAGGCTTGTAGATGCGG	224	NM_009024.2
mouse RARβ	CTCGTCCCGAGCCCACCATCTCCACTT	GAGGTCGTCTAGCTCCGCTGTCATCTC	351	NM_001289761.1
mouse RARγ	CTCATCACCAAGGTCAGCAAAGCC	CAGCCCATCCGAGAATGTCATAGT	312	NM_011244.4
mouse RPLP0	CAGGCTTTAGGCATCACCACT	GGGGGAGATGTTCAGCATGTT	132	NM_007475.5
human LOXL4 promoter DR5-1	GAGAGGTACCTCAGCATGAGAGGTGGCTGG	GAGAGCTAGCGGAGGCAGATGTGCAAGTAAG	351	
human LOXL4 promoter DR5-2	GAGAGGTACCCCGTGGCCCTTGTCAATTAC	GAGAGCTAGCTCACAACTTTCCACTCCTCGG	379	
human LOXL4 promoter mutant DR5-1	GGTGGGGCAGTATAACTGGGGGGA	TCCCCCCAGTTATACTGCCCCACC		
human LOXL4 promoter mutant DR5-2	CCCAGGCATGTATAAGCCACAGGA	TCCTGTGGCTTATACATGCCTGGG		
