# Supplementary material for: Lysyl oxidase-like 4 involvement in retinoic acid epithelial wound healing
Source: Sci Rep. 2016 Sep 6;6:32688. doi: 10.1038/srep32688 (PMC5011693; doi:10.1038/srep32688)
Supplement: Supplementary Information [file srep32688-s1.pdf]

SUPPLEMENTARY INFORMATION

* * * * *

Lysyl oxidase-like 4 involvement in retinoic acid epithelial wound healing

Aurélie Comptour ; Marion Rouzaire ; Corinne Belville ; Nicolas Bonnin ;
Estelle Daniel ; Frédéric Chiambaretta ; Loïc Blanchon ; Vincent Sapin

		T12	T36	T60	*P value*
LOX	Fold change	0,87	0,84	0,89	NS
	SD	0,1	0,12	0,06	
LOXL1	Fold change	1,08	0,92	0,93	NS
	SD	0,11	0,03	0,02	
LOXL2	Fold change	0,93	0,86	0,83	NS
	SD	0,09	0,11	0,15	
LOXL3	Fold change	1,13	0,91	0,84	NS
	SD	0,32	0,14	0,1	

LOX family members RNA expressions in HCE cells treated for 12, 36 and 60 h with atRA normalized by the geometric mean of three housekeeping genes (*n* = 5 experiments each conducted in duplicate), Mann-Whitney *U*-test, ns: no significant

Supplementary data 1 *(Comptour et al. 2016)*

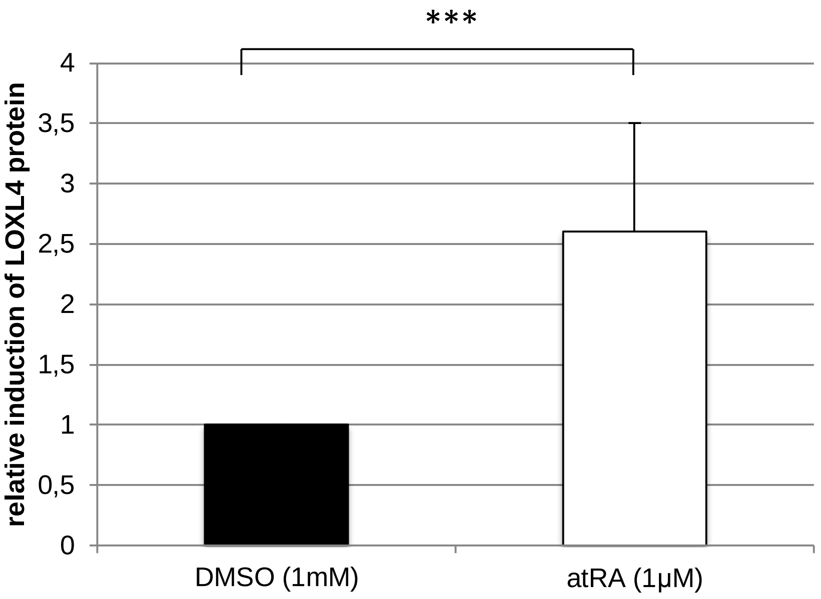

LOXL4 protein induction confirmed by Elisa test in HCE cells treated for 48 h with atRA and expressed as a ratio to DMSO (n = 3 experiments each conducted in triplicate). Each bar shows mean ± SD. Mann-Whitney *U*-test; ***: $p < 0.001$.

Supplementary data 2 (Comptour *et al.* 2016)

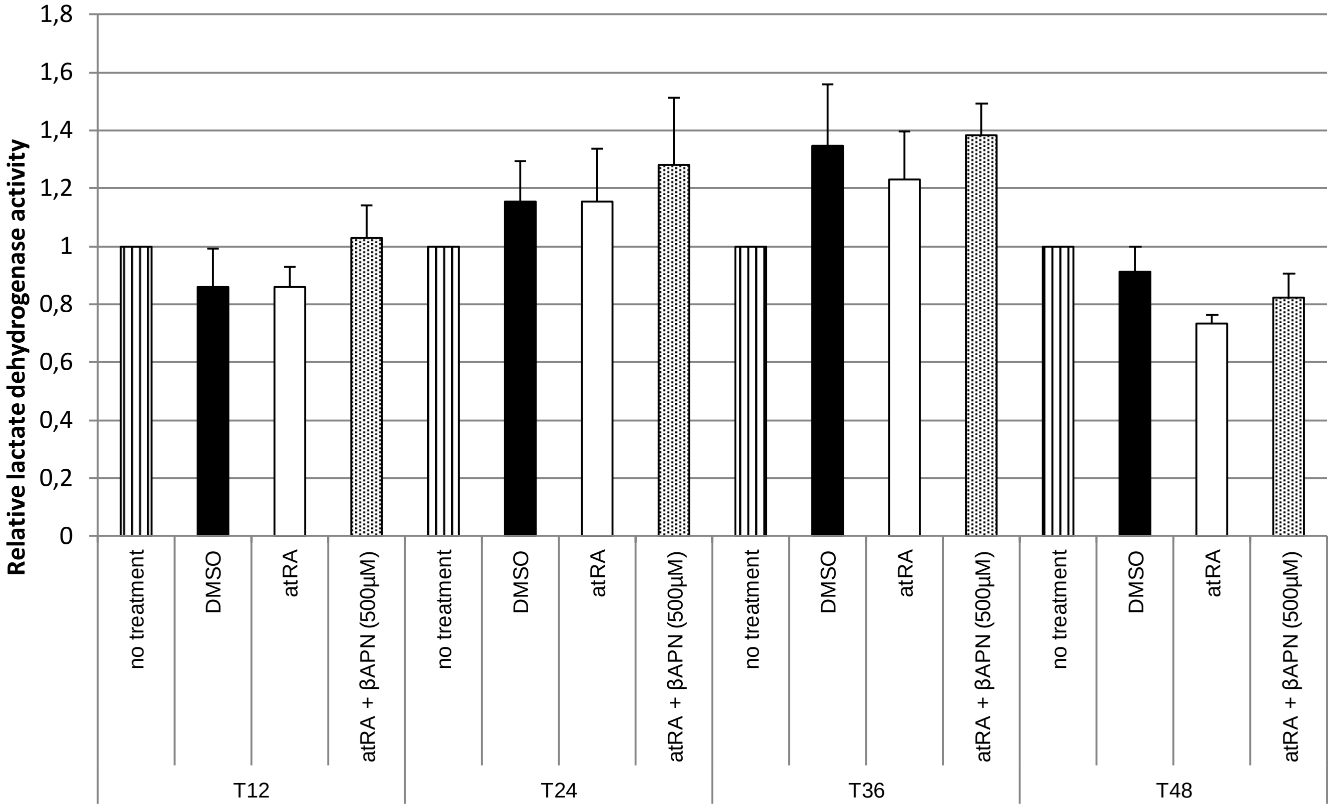

Lactate dehydrogenase (LDH) activity measured on HCE cells treated for 48 h with atRA and expressed as a ratio to no treatment sample (*n* = 3 experiments each conducted in duplicate) . These data were no significant

Supplementary data 3 (Comptour *et al.* 2016)

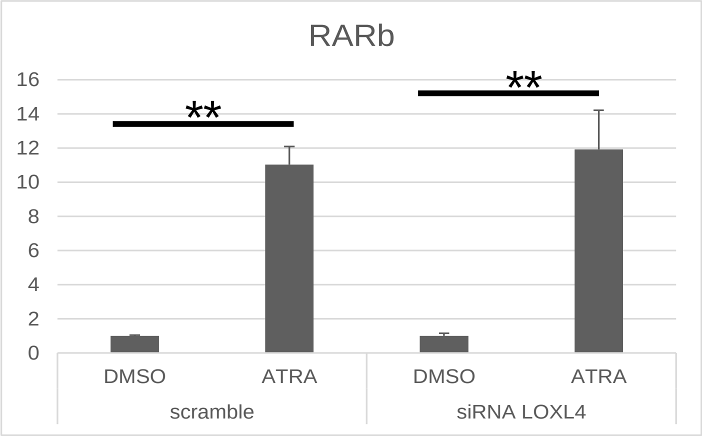

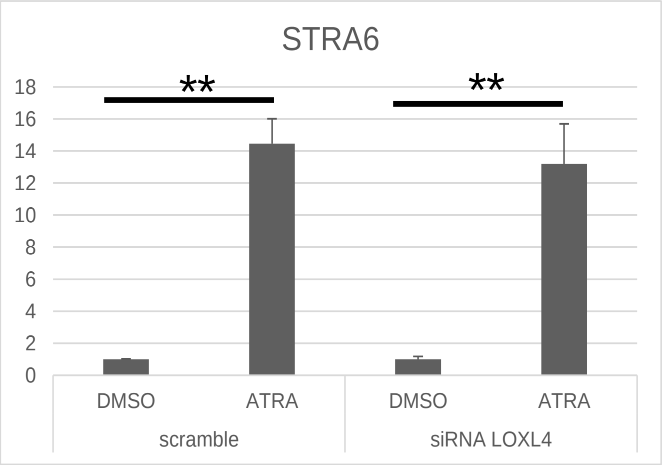

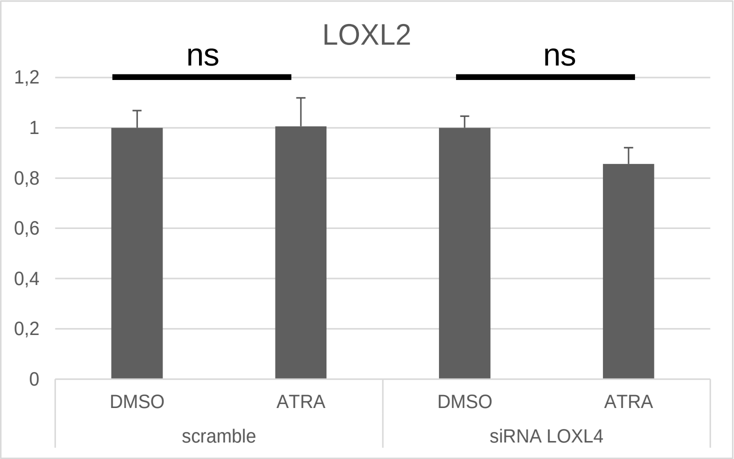

RARβ, STRA6 and LOXL2 mRNA expression in HCE cells transiently transfected by a siRNA control (scramble) or siRNA against LOXL4 and treated for 48 h with atRA. Results were normalized by the geometric mean of three housekeeping genes, and expressed as a ratio to DMSO (*n* = 3 experiments each conducted in duplicate) (**: $p < 0.01$

<u>Supplementary data 4</u> (Comptour *et al.* 2016)

GeneName	GeneSymbol	Log fold change	accesion number
lysyl oxidase-like 4	LOXL4	2,154208	NM_032211
sparc/osteonectin, cwcv and kazal-like domains proteoglycan (testican) 2	SPOCK2	5,6031632	[NM_000231]
retinoic acid receptor responder (tazarotene induced) 1	RARRES1	2,2849648	[NM_002888]
mucin 4, cell surface associated	MUC4	1,4780256	[NM_018406]
ADAM metallopeptidase with thrombospondin type 1 motif, 14	ADAMTS14	1,42365592	[NM_139155]
UDP-N-acetyl-alpha-D-galactosamine:polypeptide N-acetylgalactosaminyltransferase 12 (GalNAc-T12)	GALNT12	1,78495976	[NM_024642]
cartilage oligomeric matrix protein	COMP	2,11332512	[NM_000095]
stimulated by retinoic acid gene 6 homolog (mouse)	STRA6	3,04795072	[NM_001142617]
mucin 20, cell surface associated	MUC20	1,51305768	[NM_001098516]
desmoglein 1	DSG1	-1,36725448	[NM_001942]
adrenergic, alpha-1B-, receptor	ADRA1B	1,753744	[NM_000679]
inhibitor of DNA binding 1, dominant negativ e helix-loop-helix protein	ID1	1,49004936	[NM_002165]
SRY (sex determining region Y)-box 4	SOX4	1,29449576	[NM_003107]
dehydrogenase/reductase (SDR family) member 3	DHRS3	2,38155328	[NM_004753]
aldehyde dehydrogenase 1 family, member A3	ALDH1A3	2,40455744	[NM_000693]
keratin 13	KRT13	2,86285056	[NM_002274]
inv olucrin	IVL	1,96852608	[NM_005547]
plasminogen activ ator, tissue	PLAT	1,64096624	[NM_000930]
thrombospondin 1	THBS1	-1,22931176	[NM_003246]
neurotrophic tyrosine kinase, receptor, type 3	NTRK3	-1,3019848	[NM_001007156]
E74-like factor 3 (ets domain transcription factor, epithelial-specific)	ELF3	2,17134	[NM_004433]
tripartite motif-containing 63	TRIM63	1,2653708	[NM_032588]
serum depriv ation response	SDPR	1,2688832	[NM_004657]

The table completes the figure 2b by compiling gene name, gene symbol, log fold change and accession number of all genes classified according their physiological pathways using Genomatic®.

<u>Supplementary data 5</u> (Comptour et al., 2016)
